# Acute Presentation of Mesoamerican Nephropathy in an Agricultural Worker From Panama

**DOI:** 10.7759/cureus.38103

**Published:** 2023-04-25

**Authors:** Karen Courville, Efrain Castillo, Norman Bustamante, Rolando Millord

**Affiliations:** 1 Nephrology, Hospital Gustavo Nelson Collado, Chitre, PAN; 2 General Medicine, Universidad Latina de Panama, Panama City, PAN; 3 Pathology, Complejo Hospitalario Doctor Arnulfo Arias Madrid, Panama City, PAN

**Keywords:** chronic kidney disease of unknown etiology, chronic kidney disease, heavy metals, agrochemicals, dehydration, tubulointerstitial nephritis

## Abstract

Mesoamerican nephropathy (MeN) is Central America's growing endemic renal disorder. No single cause is established, but many risk factors are hypothesized, such as young and medium-aged adults, male sex, work environment, heavy metals and agrochemicals exposure, occupational heat stress, nephrotoxic drug use, and low socioeconomic status. The diagnosis is confirmed by renal biopsy with chronic tubular atrophy and tubulointerstitial nephritis. If biopsies are unavailable, MeN is clinically suspected in patients residing in hotspot regions with a reduced estimated glomerular filtration rate (eGFR) and the absence of defining etiology, such as hypertension, diabetes, or glomerulonephritis. Currently, there is no specific treatment for which early diagnosis and intervention on risk factors is the primary strategy to improve prognosis. We report a case of a young male with agricultural labor exposure who presented with acute abdominal pain, back pain, and renal dysfunction that later progressed to chronic kidney disease (CKD) due to MeN. This case is significant because, although MeN is well-described in the literature, few cases of acute presentation have been documented.

## Introduction

Mesoamerican nephropathy (MeN) is rising in endemic areas like Central America and South Asia. [1]. Despite being reported for the first time two decades ago, its specific cause has yet to be elucidated entirely [2]. A multifactorial etiology is proposed that includes heat stress, dehydration, and exposure to pesticides and agrochemicals [3]. MeN is not directly related to diabetes mellitus, hypertension, glomerulonephritis, or other known causes of chronic kidney disease (CKD) [4]. Most patients are asymptomatic but can present with symptoms of advanced CKD, such as recurrent nausea, vomiting, back pain, chronic muscle cramps, weakness, and arthralgias [5]. The most common histopathological findings are tubulointerstitial nephritis in the early stage, segmental sclerosis associated with rapidly deteriorating kidney function, and interstitial fibrosis in later stages [6]. No specific treatment exists; only preventive measures have shown a favorable long-term prognosis [7]. Poor healthcare access and other social risk factors have been associated with a poor prognosis due to a delayed diagnosis [8]. We present a case of a patient with an acute presentation of MeN that later progressed to chronic MeN. We aim to raise awareness of this entity's acute and chronic presentation.

## Case presentation

A 44-year-old male patient comes for evaluation due to one month of intermittent colicky abdominal pain associated with reduced appetite, intermittent headache, and back pain. During the initial evaluation, a baseline serum creatinine of 3.04 mg/dL (normal 0.6-1.2 mg/dL) was found, prompting a reference to the Nephrology department due to suspected acute kidney injury. No background history or findings of hypertension or diabetes mellitus were present on admission. The patient's medical history includes occasional non-steroidal anti-inflammatory drugs (NSAIDs) with a mean of three tablets a week, and denies using other medications, smoking, or illicit drugs. However, the patient was found to have a history of alcohol use lasting 15 years, with a mean alcohol consumption of 5-7 drinks/day (1 drink = 14 g ethanol). His family medical history was negative for kidney disease. The patient has worked as an agricultural field laborer for 25 years in different crops (tomatoes, peppers, sugar cane). The review of symptoms was negative for vomiting or diarrhea but positive for bilateral lower limb muscle weakness, decreased appetite, and adynamia for one month. Examination revealed stage II arterial hypertension (148/90 mm of Hg), low-grade fever (37.6º Celsius), BMI of 22.31 kg/m2, heart rate of 96 beats per minute, well-hydrated oral mucosa, and soft and non-tender abdomen with normal bowel sounds. Bilateral hand swelling and mild tenderness on palpation of the metacarpophalangeal and proximal interphalangeal joint were found. Lower extremities did not show edema. Chest auscultation revealed fine basilar crackles bilaterally. Electrocardiography (ECG) and posteroanterior (PA), anteroposterior (AP), and lateral view chest X-rays (CXR) did not show abnormalities. Important laboratory values on admission include serum creatinine of 3.04 mg/dL, blood urea nitrogen (BUN) of 38 mg/dL, and a uric acid concentration of 8.8 mg/dL (Table 1). Urinalysis showed 20 leukocytes per high power field and 220 mg/g of urine protein/creatinine ratio (Table 2).

**Table 1:** Main laboratory data on admission. Hb: Hemoglobin; MCV: Mean corpuscular volume; ESR: Erythrocyte sedimentation rate; SCr: Serum creatinine; BUN: Blood urea nitrogen; Na+: Sodium; K^+^: Potassium; Ca^2+^: Calcium; P: Phosphorus; Mg2+: Magnesium; Alb: Albumin; TC: Total cholesterol; HDL: High-density lipoprotein; LDL: Low-density lipoprotein; TG: Triglycerides.

Laboratory values	On admission	Normal range
Hb	12.9	13.5-17.5 g/dL
MCV	86	80-100 μm^3^
Leukocyte count	9.32	4,500-11,000/mm^3^
Platelet count	288	150,000-400,000/mm^3^
ESR	45	0-15 mm/h
Glucose	119	70-110 mg/dL
SCr	2.89	0.6-1.2 mg/dL
BUN	38	7-18 mg/dL
Uric acid	8.8	3.0-8.2 mg/dL
Na^+^	137	136-145 mEq/L
K^+^	3.7	3.5-5.0 mEq/L
Ca^2+^	8.7	9.0-10.5 mg/dL
P	3.0	3.0-4.5 mg/dL
Mg^2+^	1.6	1.5-2.4 mg/dL
Alb	3.7	3.5-5.5 g/dL
TC	187	< 200 mg/dL
HDL	44	40-60 mg/dL
LDL	78	< 160 mg/dL
TG	75	< 150 mg/dL

**Table 2:** Urinalysis and urine chemistry. HPF: High power field.

Test	Result	Reference
Color	Yellow	
Aspect	Clear	
Specific gravity (SG)	1.015	1.005-1.035
pH	6.0	5.0-8.0
Blood	1+	Negative
Leukocytes	20/HPF	0-5
Glucose	Negative	Negative
Proteins	2+	Negative
Ketones	Negative	Negative
Urine protein/creatinine	220 mg/g	0-20

**Figure 1:**
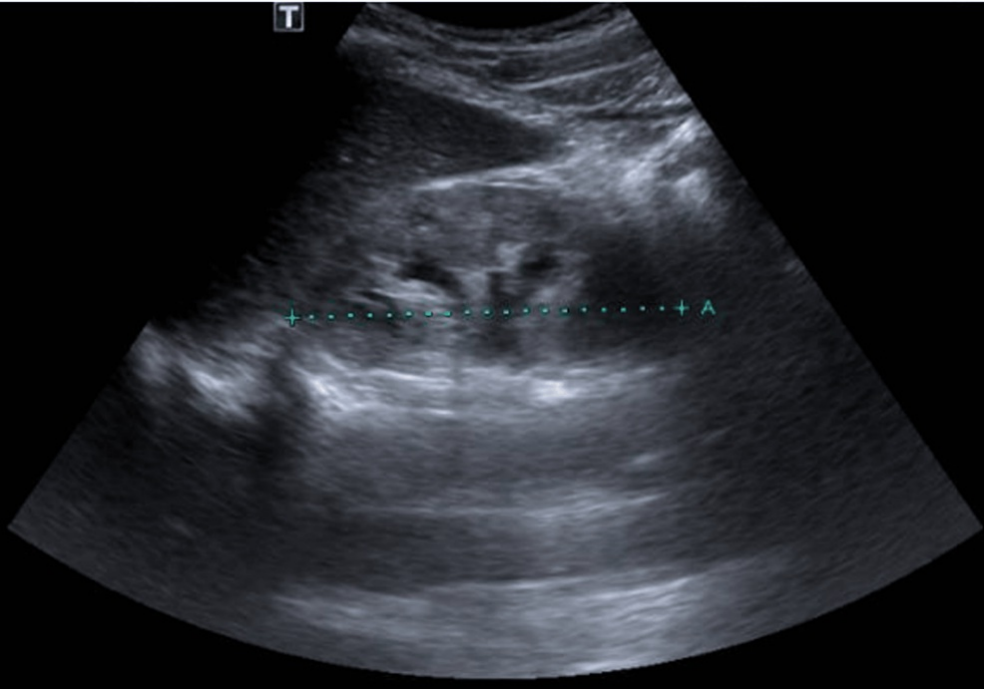
Renal ultrasound. Right kidney 9.6 cm, left kidney 9.1 cm long; no hydronephrosis, increase in echogenic pattern.

Immunologic and viral studies including hepatitis B surface antigen (HBsAg), hepatitis B type e antigen (HBeAg), total antibody to hepatitis B core antigen (anti-HBc), hepatitis C virus antibody (Anti-HCV), HIV, the venereal disease research laboratory (VDRL) test, antinuclear antibodies (ANA), anti-double stranded DNA (anti-dsDNA), and the antineutrophil cytoplasmic antibodies (ANCAs), were all negative. Other diagnostic workups, such as kidney ultrasound (Figure 1), glycated hemoglobin (HbA1c), complement component three (C3) and four (C4), and arterial blood gas (ABG), did not yield causative factors for kidney dysfunction.

**Figure 2:**
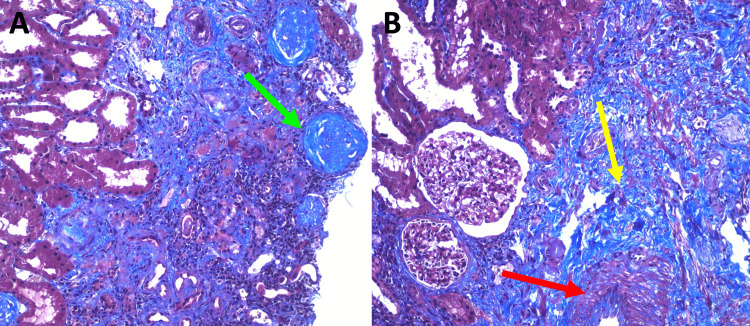
Renal biopsy using Masson's trichrome stain. Tubulointerstitial fibrosis (70% of tissue sample) in blue (yellow arrow), and mild sclerosis of the intima (red arrow). Some glomeruli are globally sclerosed (green arrow). No crescents or immune deposits were found in light microscopy.

During hospitalization, he was treated with a 0.9% saline solution infusion at 60 mL/hour for 48 hours, with a total of 2.88 L received and a urine output of 1.2 mL/kg/hour. The patient was treated with allopurinol 150 mg daily to manage the hyperuricemia. The renal biopsy revealed extensive areas (70% of the tissue sample) of tubular atrophy, interstitial fibrosis, and mild arterial sclerosis of the intima (Figure 2). No deposits were found on immunofluorescence, and electron microscopy was not available.

Upon discharge, allopurinol 150 mg daily was maintained for three months. An outpatient referral to Nephrology for follow-up, alcohol abstinence counseling, and lifestyle change recommendations such as adequate hydration, NSAID avoidance, and a low-purine diet were provided. Follow-up at three months revealed a 0.5 mg/dL increase in serum creatinine from baseline. Follow-ups at six and nine months revealed normalization of uric acid levels, glucose within normal values without treatment, and creatinine values at ​​around 2.7 mg/dL. Blood pressure remained under 140/90 mmHg without antihypertensive therapy.

## Discussion

MeN has become a more prevalent and concerning clinical entity for the past 20 years [9]. Furthermore, there has been a fast-growing worldwide rate of CKD cases, with a relatively higher number of cases not having identifiable etiology [1, 9]. The most common causes of CKD are systemic diseases such as diabetes mellitus, hypertension, and primary or secondary renal disorders, classified as traditional causes of CKD. However, MeN is categorized as a non-traditional cause of CKD [10]. The pathogenesis is not entirely understood, but studies have contributed to elucidating the pathophysiology by describing that MeN mainly affects the working population with heavy tasks, such as fieldworkers where high temperatures are handled, either by heat stress due to excessive sun exposure and high temperatures or dehydration induced by the activity [11-13]. In addition, these patients have a history of NSAIDs overuse [11] and alcohol consumption [14]. Male farmers carry a double burden of non-traditional (occupational, environmental, toxic) and epigenetic risks that augment the development of tubular and interstitial damage. Most patients are young or middle-aged adults, otherwise-healthy patients with good physical conditions [15]. Some clinical manifestations include nausea, lower back pain, and abdominal discomfort, but since they seem related to their work, they often do not consult for these symptoms. In chronic cases, patients report muscle weakness, arthralgia, and muscle cramps [5]. The diagnosis of MeN can be classified as suspicious, probable, or confirmed. A suspicious diagnosis can be made in a patient with a decrease in eGFR below 60 mL/min/m2, mild proteinuria (non-nephrotic), absence of autoimmune disorder, glomerulopathies, congenital kidney disease, obstructive renal disease, or polycystic kidney disease [16]. Tubular atrophy and interstitial fibrosis with some capillary sclerosis are classical findings in MeN. Glomerular architecture and GBM sclerosis are seen in the later stages, with disease progression [1]. The treatment of MeN is supportive and requires evaluation and follow-up as that of CKD from any other cause. Electrolyte monitoring is mandatory; potassium, magnesium, and sodium supplements must be used if necessary. Advanced CKD due to MeN is managed with renal replacement therapy [17]. Although renin-angiotensin-aldosterone system (RAAS) blockers are renoprotective in other forms of CKD, RAAS blockade is not advised in patients with MeN due to the increased risk of acute kidney injury in the setting of hypovolemia [18]. Because preventive measures are the only strategies to improve long-term prognosis [7], it is critical to reduce the triggers as much as possible by reducing heat exposure by encouraging personal protective equipment use, controlling work schedules and providing shaded areas, avoiding dehydration, reducing agrochemicals and heavy metals exposure, avoiding NSAIDs and nephrotoxic drugs, limiting the intake of sugary and alcoholic drinks, and providing clean water access, among others. Early diagnosis and follow-up are essential in delaying or avoiding progression.

It is important to note that there have been no rigorous studies investigating the early stages of MeN. Current case reports have focused on risk factors, chronic symptoms, hyperuricemia, and histopathological findings. However, no early disease cases have been reported progressing to the classically described chronic stage of MeN. Our case highlights the importance of this condition as a serious cause of kidney dysfunction and provides the clinical picture of nonspecific symptoms, leukocyturia, and hyperuricemia; and is the first case of MeN confirmed with a biopsy in Panama.

## Conclusions

MeN is a nontransmissible and nonproteinuric chronic tubulointerstitial disorder that typically presents in young adults and agricultural workers, predominantly in Central America. The pathophysiology is not completely known, but it is likely caused by repeated episodes of acute kidney injury (AKI) associated with heat stress, repetitive dehydration, strenuous physical activity, and environmental and occupational toxins exposure causing constant inflammation that leads to acute and chronic tubulointerstitial nephritis. Most cases occur in vulnerable populations, low-altitude and coastal regions of El Salvador, Nicaragua, Guatemala, Costa Rica, and Panama. The diagnosis is suspected in young adult patients working in tropical areas presenting with advanced kidney disease and subsequent biopsy showing tubular atrophy, interstitial nephritis, and segmental glomerulosclerosis. In developing countries or hospitals with limited resources, MeN can be established clinically using accepted clinical criteria, positive history of occupational risk, and the absence of traditional causes of CKD. Since there is no specific treatment, the most important therapeutic approach is to prevent precipitating events and manage CKD-related complications on time. People at risk of MeN should drink adequate fluids that contain sufficient amounts of sodium and potassium, limit exposure to heat, and avoid nephrotoxic drugs. The education of patients and healthcare members is critical to achieving complete management while the pathogenesis and therapeutic measures are being investigated.
